# Actin-interacting and flagellar proteins in *Leishmania spp*.: Bioinformatics predictions to functional assignments in phagosome formation

**DOI:** 10.1590/S1415-47572009000300033

**Published:** 2009-09-01

**Authors:** Michely C. Diniz, Marcília P. Costa, Ana C. L. Pacheco, Michel T. Kamimura, Samara C. Silva, Laura D. G. Carneiro, Ana P. L. Sousa, Carlos E. A. Soares, Celeste S. F. Souza, Diana Magalhães de Oliveira

**Affiliations:** Núcleo Tarcísio Pimenta de Pesquisa Genômica e Bioinformática, Universidade Estadual do Ceará, Fortaleza, CEBrazil; 2 Laboratório de Imunomodulação e Protozoologia, Fundação Oswaldo Cruz, Manguinhos, RJBrazil

**Keywords:** actin-interacting proteins (AIPs), flagellar proteins, *Leishmania*, coronin and Arp2/3, phagosome

## Abstract

Several motile processes are responsible for the movement of proteins into and within the flagellar membrane, but little is known about the process by which specific proteins (either actin-associated or not) are targeted to protozoan flagellar membranes. Actin is a major cytoskeleton protein, while polymerization and depolymerization of parasite actin and actin-interacting proteins (AIPs) during both processes of motility and host cell entry might be key events for successful infection. For a better understanding the eukaryotic flagellar dynamics, we have surveyed genomes, transcriptomes and proteomes of pathogenic *Leishmania spp*. to identify pertinent genes/proteins and to build *in silico* models to properly address their putative roles in trypanosomatid virulence. In a search for AIPs involved in flagellar activities, we applied computational biology and proteomic tools to infer from the biological meaning of coronins and Arp2/3, two important elements in phagosome formation after parasite phagocytosis by macrophages. Results presented here provide the first report of *Leishmania* coronin and Arp2/3 as flagellar proteins that also might be involved in phagosome formation through actin polymerization within the flagellar environment. This is an issue worthy of further *in vitro* examination that remains now as a direct, positive bioinformatics-derived inference to be presented.

## Introduction

*Leishmaniaspp.* is a trypanosomatid protozoan responsible for a complex group of diverse clinical forms of classically neglected diseases collectively known as leishmaniases (Peters and Killick-Kendrick, 1987). As an insinuating and persistent pathogen, *Leishmania* has a specialized organelle for motility, the flagellum, which is essential for parasite migration, invasion and persistence on host tissues. Several motile processes have been reported in the literature that could be responsible for the movement of proteins into and within the flagellar membrane (Kozminski *et al.*, 1993). However, little is known about the process by which specific proteins (either actin-associated or not) are targeted to protozoan flagellar membranes, with few exceptions such as the work by Snapp and Landfear (1999) which targets a motif for a flagellar integral membrane protein in *Leishmania enriettii.* Actin is a major cytoskeleton protein, while polymerization and depolymerization of parasite actin and actin motor-associated proteins during both processes of motility and host cell entry might be key events for successful infection.

To gain a better understanding of flagellar dynamics in *Leishmania*, we have surveyed genomes, transcriptomes and proteomes of these pathogens to identify pertinent genes/proteins and to build *in silico* models to properly address their putative roles in trypanosomatid virulence. We have applied computational tools (hidden Markov models, Viterbi algorithm and comparative modeling) to infer biological meaning through detailed sequence-structural-functional analyses on actin-interacting proteins (AIPs) involved in flagellar activities of *Leishmania spp*. The actin system evolved to be fail-safe with multiple proteins sharing overlapping but novel roles, whereas these multifaceted roles are likely to provide the versatile scenario involving actin-interacting activities.

Proteins such as coronin and Arp2/3 complex have a common feature of actin-binding activity and might be involved in *Leishmania* intraflagellar pathways (Broadhead *et al.*, 2006; Costa *et al.*, 2007). The coronin family comprises two groups of evolutionary conserved WD-repeat proteins known to interact with Arp2/3 complex that help regulate the nucleation dynamics of actin filaments (Rybakin and Clemen, 2005). Invasion and differentiation processes in *Leishmania* directly involve phagocytosis and the formation of an intracellular membrane-bounded organelle called a phagosome, since *Leishmani*a promastigotes must first evade complement-mediated lysis until they are engulfed by a mammalian host macrophage (Puentes *et al.*, 1989; Olivier *et al.*, 2005).

By phagocytosis we mean the process of ingestion per se. A phagosome is the endocytic compartment that contains a non-interfering particle, as opposed to a vacuole which describes a compartment containing a particle, such as a pathogen like *Leishmania* that diverts normal phagosome maturation. Phagosome maturation refers to the process of intracellular phagosome development after closure of the phagocytic cup (reviewed by Haas, 2007). Phagocytosis has evolved into a highly complex and regulated process in multicellular eukaryotes by which microbes and other particles are taken up into the phagosome (Griffiths and Mayorga, 2007). The precise mechanisms by which coronin and Arp2/3 contribute to phagocytosis are not known (Yan *et al.*, 2005), but it is crucial to investigate elements that will shed more light on these mechanisms.

Although not clearly understood, it has been shown *in vitro* that, after the phagocytosis of *L. donovani* by macrophage-like cells J774, parasites are transiently located in phagosomes with poor fusogenic properties towards late endocytic compartments (Desjardins and Descoteaux, 1997). In contrast, after internalization, they are found in compartments that rapidly fuse with late endocytic organelles (Dermine *et al.*, 2000). A possibility is that these distinctive features of the early phagosomes could be linked to different proteins expressed on the parasite surface, which may modify the fusion capacity of the phagosomal membrane (Desjardins and Descoteaux, 1997; Dermine *et al.*, 2000). Furthermore, most of the *Leishmania*-containing phagosomes have been shown to accumulate F-actin, which is noted around these phagosomes (Holm *et al.*, 2001).

We should recall, then, the early events following phagocytosis of *Leishmania*, pre-adapted to the encounter within intracellular conditions of mammals. As reported (Courret *et al.*, 2002), young phagosomes containing *Leishmania* rapidly acquire a competence to fuse with late endosomes/lysosomes. If we take this version, AIPs have a greater chance of being key elements on the phagosome maturation than any of the many surface membrane proteins that have been widely investigated. For that reason, we have focused our studies on AIPs because they are rich in motif-binding activities, besides being excellent models for sequence and structural comparisons, predictions and examinations to be carried out with all kinds of restraints on spatial structure of the amino acid sequence(s) and ligands. Our coronin and Arp2/3 model restraints were derived from known related protein structures (comparative modeling) and from rules of secondary structure packing (combinatorial modeling). Our *in silico* examinations provide information about AIPs in the specific domain of phagosome function in *Leishmania spp.* infection. The role of the phagosome is to deliver particulate material to a hydrolytic environment that will lead to its degradation. The maturation process of the phagocytic compartment is linked intimately to both digestive housekeeping processes and innate sensing of molecules associated with infection (Vieira *et al.*, 2002). Therefore, mechanisms and regulation behind this maturation process are strategic for both hosts and pathogens, whereas here we present and discuss results concerning *Leishmania* AIPs that are also flagellar proteins that might be targeted in phagosome formation.

## Methods

###  Biological databases and bioinformatics tools

As previously (Oliveira *et al.*, 2005; Gouveia *et al.*, 2007; Vasconcelos *et al.*, 2007, 2008), we have used publicly available datasets of individual/clusters of gene/protein data on *Leishmania spp*. (from GeneDB, a core part of Sanger Institute Pathogen Genomics) and from other available eukaryotic organisms (at NCBI and UniProt/Swiss-Prot/trEMBL knowledge DB), including sequences from the genome projects of *C. reinhardtii* in the U.S. Department of Energy (JGI) and all available data at The *Chlamydomonas* Flagellar Proteome Project. For databases (DB) searches, accession numbers/identifiers are those used in four main sources: NCBI, GeneDB, PDB and UniProt. As previously described (Oliveira *et al.*, 2005; Gouveia *et al.*, 2007; Vasconcelos *et al.*, 2007, 2008), BLAST and its variants (Altschul *et al.*, 1997) and MUSCLE (Edgar, 2004) were used for sequence similarity searches and comparisons/analysis through pairwise and multiple alignments. As previously detailed (Costa *et al.*, 2007), for pattern recognition tasks of gene/protein predictions, motif finding and refinements for defining core domains of interest, HMM implementations such as HMMER (Eddy, 1998) and a structural prediction adaptation (Yoshizawa *et al.*, 2006) were employed, with the additional improvement of the Viterbi algorithm (VA) (Forney, 1973; Black, 2004) for best possible alignments after recursion (Alexandrov and Gerstein, 2004). As previously (Costa *et al.*, 2007), we assume that the HMMs generated the input sequence and are looking for the highest probability path. For sequence position *i* = 0, 1 ,…, *L+1*; for state *l* = 0, 1, …, *n*:



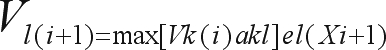


### *In-silico* survey

**Figure 1:**
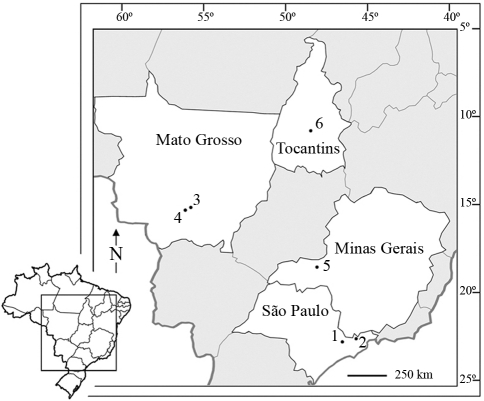
Bioinformatics methods employed in this work, shown as a briefly simplified scheme of tools to illustrate a stepwise process in biological information technology.

As described in previous studies (Oliveira *et al.*, 2005; Gouveia *et al.*, 2007; Vasconcelos *et al.* 2008), we took alignments created with FASTA/BLAST as input and computed alignment tables, providing hierarchical and successive correlations between each of the two sets of sequences. FASTA files for amino-acid (aa) sequences of coding regions were downloaded from sources detailed above. All flagellar proteins and AIPs in *Leishmania* were collected from experimental papers and from the comprehensive GeneDB database. An illustration of the tools used is seen in Figure 1. Briefly, these sequences were used as queries against genomic datasets with PSI-BLAST (BLASTP2.2.10) (Altschul *et al.*, 1997) to determine sequence similarity among all possible sequences, followed by multiple sequence alignments (MSA) performed with MUSCLE (Edgar, 2004) on target entries of main source DBs searched against various collections of protein motifs and families. Results of MSA were used as training datasets for HMM profiles (Eddy, 1998; Yoshizawa *et al.*, 2006) and the SMART (Letunic *et al.*, 2006) motif patterns of cluster motifs. The quality of trained HMM predictions and SMART pattern matching was examined. The HMM profiles for cluster motifs were trained, calibrated, and used for DB searches using HMMER package ver.2.3.1 with default parameters. Gene ontology (GO) terms were assigned, based on top matches to proteins with GO annotations from Swiss-Prot/trEMBL, AMIGO after GeneDB and TargetP access. Functional assignment of these genes/gene products was inferred using an RPS-BLAST search against conserved domain DBs (CDD) (Marchler-Bauer *et al.*, 2005); information was taken into account about subcellular localization (Emanuelsson *et al.*, 2000), sequence and structural features, domains/motifs conservation (von Mering *et al.*, 2005; Letunic *et al.*, 2006**),** and *in vitro* characterization (Avidor-Reiss *et al.*, 2004; Tull *et al.*, 2004). For three-dimensional (3D) modeling of AIPs we employed Modeller (Sali and Blundell, 1993; Marti-Renom *et al.*, 2000) and ESyPred3D (Lambert *et al.*, 2002). For visualization of 3D modeled structures, we employed the browser plug-in Chime. For addressing particular sites of structural-functional relationships of AIPs we used Cn3D (NCBI) and STING (Higa *et al.*, 2004). Best models were evaluated by using PROCHECK (Laskowski *et al.*, 1993) and a thorough analysis of consensus AIPs structures was carried out for model dissection, superposition and rms deviation (rmsd) calculations.

###  Proteomic analyses

*Samples.* Purified fractions of flagella from *Leishmania amazonenesis*, strain H21 (MHOM/BR/76/ MA-76), were kindly provided by Fiocruz (Fundação Oswaldo Cruz, Manguinhos, Rio de Janeiro). An amount of 250 μg of flagellar protein extract was normalized for use.

*Two-dimensional (2D) gel electrophoresis*. We performed proteomic analyses through 2D polyacrylamide gel electrophoresis (2D-PAGE), as previously described (Drummelsmith *et al.*, 2003, Brobey *et al.*, 2006). Briefly, proteins were treated with a lysis buffer with 7 M urea, 2 M thiourea, 2% NP-40, 2% DTT and 2% ampholytes (pH 3 to 10). In the first dimension, a protein separation by isoelectric point was performed applying IPG strips, soaked in the sample solution, at 300V for 5 min, 3,500 V for 5 h through Ettan *IPGphor II* Isoelectric Focusing System (GE Healthcare). To promote efficient protein transfer from the first to the second dimension, IPG strips were incubated in reducing buffer (1% DTT, 6M urea, 30% glycerol, 2% SDS, 50 mM Tris, pH 8.8) for 15 min, followed by 15 min incubation in alkylation buffer (2.5% iodoacetamide, 6 M urea, 30% glycerol, 2% SDS, 50 mM Tris, pH 8.8). The strips were immediately applied to the second dimension 12.5% polyacrylamide gel. Strips were, then, overlaid on freshly poured 10% Tricine-SDS gels (18 x 16 cm) and sealed with agarose solution (0.5% agarose, plus a few grains of bromophenol blue in a Tris-tricine cathode buffer). Protein standards of molecular mass range 14400 Da to 97000 Da were used (GE Healthcare). Gel electrophoresis was carried out at 250 V for 1 h, and then at 500 V until the dye front reached the bottom of the gel (around 8 h). Gels were silver-stained as previously described (Gromova and Celis, 2006).

After running and scanning the gels, the resulting protein gel was analyzed using ImageMaster 2-D Platinum 6.0^®^ (Amersham Biosciences, Uppsala, Sweden). The spots detected automatically by the software were visually inspected. Spot filtering and editing were performed manually to remove artifacts and to correct for spots that did not split correctly or were not detected by the software's automatic spot detection process. Molecular mass (MW) and isoeletric point (pI) were predicted for the best spots.

## Results

To identify putative flagellar proteins of *Leishmania* participating in the actin system, we have taken into account all actin-binding, interacting or related/regulated proteins as source data to investigate their possible involvement in assembly of F-actin (the principal driving force behind many forms of parasite locomotion, including those regarding or underlying internalization into the host cell). Our underlying hypothesis is that polymerization and depolymerization of parasite actin and actin motor-associated proteins, during both processes of motility and host cell entry, might be key events for successful infection, including the parasite survival within phagosomes. Recently (Vasconcelos *et al.*, 2007, 2008) we reported an actin-polymerization *Leishmania* protein, profilin, and its partner, formin, as putative flagellar proteins due to their likely involvement in axonemal assembly/disassembly, therefore, flagellar dynamics/remodeling. In addition, we predicted other *Leishmania* AIPs, coronins and Arp2/3 complex as being flagellar proteins after detailed *in silico* structural alignments (Costa *et al.*, 2007). Now, we focus on *Leishmania* coronin and Arp2/3 complex proteins as flagellar elements to phagosome formation in an attempt to directly associate the flagellum to the phagosomes, which are pivotal organelles in the ability of macrophages to perform several of their key functions, such as the handling of apoptotic cells, tissue remodeling, and restriction of the establishment and spread of intracellular pathogens (Méresse *et al.*, 1999), such as *Leishmania*. Comparative bioinformatics analyses (performed after *in silico* genomic searches and *in vitro* proteomic screenings) have led to the identification of flagellar proteins that are involved not only in cytoskeleton activity, signaling, endocytosis, and lytic activity, but also putatively in the phagosome formation. With this study, we are able to confirm previous data on proteins already shown to be involved in actin systems, but not previously reported as *Leishmania*- or as flagellar-associated.

###  Flagellar coronin identification in *Leishmania*

**Figure 2:**
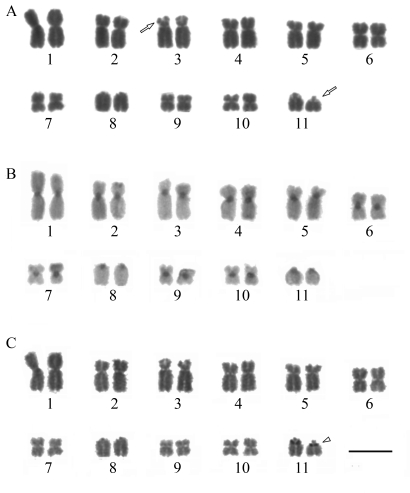
Sequence-structural alignments of four *Leishmania* coronins. a), b), c) and d): 3D models of *L. major* (LmjF23.1165/CAC44941.1), *L. infantum* (LinJ23.1360), *L. donovani* (AAY56362.1) and *L. braziliensis* (LbrM23.1230), respectively, illustrating the putative actin-binding region (N-terminus). Left-panel: schematic diagram of corresponding regions of other coronin family proteins. The first 30 amino acids are shown to illustrate residues conserved across members of the coronin family (*Trypanosoma brucei, Cryptosporidium parvum, Toxoplasma gondii, Plasmodium falciparum, Mus musculus* and *Bos taurus*).

Coronins constitute an evolutionary conserved family of WD40-repeat (WDR) actin-binding proteins (reviewed by de Hostos, 1999; Rybakin and Clemen, 2005), originally described in the social amoeba *Dictyosteliumdiscoideum* (de Hostos *et al.*, 1991). They can be divided, according to structure and function, into two subfamilies: one of shorter proteins (45-50kDa) implicated in, among other functions, nucleation of F-actin; and the other of longer, around 900 aa length, highly homologous coronins (POD-1 and Crn7) found in *C. elegans*, *Drosophila* and *D. discoideum* (Appleton *et al.*, 2006; Rybakin *et al.*, 2006). Until now, biochemical activities of coronins have been largely unknown, although one report (Humphries *et al.*, 2002) identified how coronin and Arp2/3 complex interact *in vitro*. The comparative alignment of primary aa sequences of *Leishmania* coronins showed that they are highly conserved and have similar domain structures (Figure 2a), sharing ~45% aa identity with *D. discoideum*. Our models for the predicted 3D structure of *Leishmania* coronins, seen in Figure 2b, also reinforce their close proximity to PDB solved structures for coronin.

**Figure 3:**
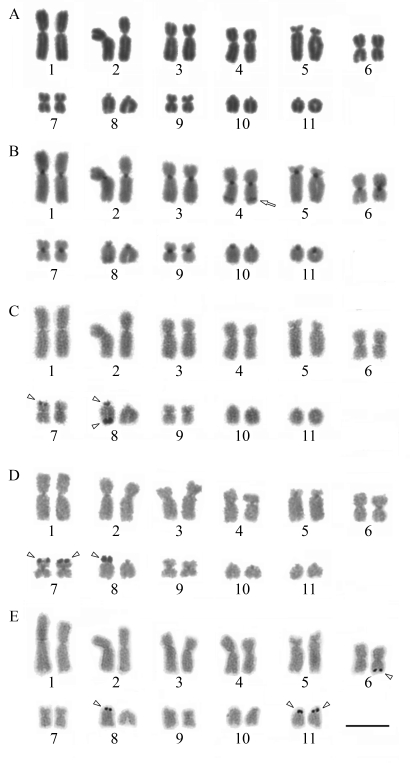
3D visualization of Arp2/3 complex modeling, including possible binding site to a coronin WDR domain at central (blue) region and multiple alignment of the amino acid sequences of Arp2/3 complex proteins from different organisms. A) PDB solved structure of *Bos taurus* Arp2/3 complex (1K8K) used as a template for building comparative models of *Leishmania* Arps with sequence from B) *L. braziliensis* (LbrM02.0360); C) *L. infantum* (LinJ02.0520) and D) *L. major* (XP_822258.1). *N-terminus; **C-terminus. E) Multiple alignment of the amino acid sequences of Arp2/3 complex proteins. Accession numbers are: *T. cruzi* (XP_810627.1), *T. brucei* (XP_951567.1), *D. rerio* (NP_991100.1), *H. sapiens* (NP_005709.1), *S. cerevisiae* (NP_012912.1), *L. infantum* (LinJ02.0520), *L. major* (XP_822258.1) and *L. braziliensis* (LbrM02.0360). Residues identical or similar are shaded dark or light gray, respectively. Dashes indicate gaps introduced for optimal alignment.

The proteomic screening performed on the flagellar fraction of *L. amazonensis* enabled us to anticipate evidence of coronin, among other proteins, as at least six putative spots, as shown in Figure 3 which provides a fairly good insight on *Leishmania* flagellar proteins (manuscript in preparation) that are also classical AIPs, such as coronin. Since coronin can be required for an early step in phagosome formation (Yan *et al.*, 2005), which is consistent with its role in actin polymerization and accumulation around phagosomes formed during the ingestion of mycobacteria (Ferrari *et al.*, 1999) and also in preventing phagosome maturation and mycobacterial killing (Ferrari *et al.*, 1999), here we have considered this information as an important element for assigning a possible phagosome role for *Leishmania* coronin as well. We must recall that *Leishmania* has differential levels of survival inside phagosomes, as has been elegantly reported by Gueirard *et al.* (2007), whose work with *L. donovani* shows degraded parasites in spacious phagosomes, in contrast to morphologically intact parasites in tight compartments within neutrophils. These results suggest that the survival of parasites is linked to their ability to be targeted to tight/non-cidal and non-degradative compartments.

It is of paramount interest to mention that a phagosome, when it normally matures into a phagolysosome, undergoes a transition that correlates with functional changes from an organelle with early endosomal characteristics to a compartment with lysosomal, degradative properties. If we consider that persistent accumulation of coronin around phagosomes containing pathogens is believed to prevent phagosome maturation and mycobacterial killing (Ferrari *et al.*, 1999), it would be conceivable to determine how coronin, actively recruited by these pathogens, may be involved in the modulation of the actin cytoskeleton, thereby influencing intracellular trafficking and survival (Jayachandran *et al.*, 2008). While broad investigations into this are ongoing, the few pieces of information available stem largely from studies on phagosomes containing pathogens such as *Leishmania* that force the maturation machinery to slow down but will not permanently inhibit phagolysosome formation, with growth dependent upon eventual phagolysosome formation (Haas, 2007).

To what extent these different killing pathways (delayed or not) are used in a given phagosome certainly depends on various cellular and environmental conditions (usually a plethora of macrophage receptors at variable proportions that may influence phagosome fate to variable degrees), including interactions with the flagellum apparatus. We must bear in mind that flagella are dynamic structures that exchange up to 20% of their polypeptides within 3 h without any changes in their overall length (Song and Dentler, 2001; Wiese *et al.*, 2003). Because flagellar microtubules are constantly turning over, the phagosomal membrane remains tightly associated with the parasites but the compartments become shorter (Marshal and Rosembaum, 2001). This reduction in size correlates with the progressive loss of the flagellum, suggesting that the parasite remodeling is accompanied by the removal of the phagosomal membrane.

Proteins involved in actin cytoskeleton dynamics that were recently implicated in phagocytosis in another protozoan, *Entamoeba histolytica*, are actobindin, coactosin and formin-like proteins (Marion *et al.*, 2005), members of the so-called “core machinery” involved in *de novo* polymerization of actin filaments, which includes several subunits of the Arp2/3 complex, CAP protein, profilin and ADF/cofilin protein family (Loisel *et al.*, 1999; Marion *et al.*, 2005). ADF/cofilin proteins are essential to activate the turnover of actin filaments in dynamic regions of the cell and have already been shown to promote the remodeling of the actin network beneath the phagocytic cup (Bamburg, 1999; Bierne *et al.*, 2001) and they have also been the subject of our own bioinformatics analyses (Pacheco *et al.*, 2007) and of a recent *in vitro* identification (Tammana *et al.*, 2008) in *Leishmania* flagellum.

Here we have chosen to follow the hypothesis that the actin filaments beneath the phagocytic cup could be organized as a bundle-like structure, rather than a dendritic network, which is necessary to form a stiff actin network around the phagocytic cup thereby facilitating the contractile activity that closes the vacuole (Marion *et al.*, 2005). It is tempting to recall the model in which *Toxoplasma gondii*-containing vacuoles possess deep invaginations supported by host microtubules that deliver host endosomes and lysosomes into the parasitophorous vacuole (Coppens *et al.*, 2006), where the parasites feed on these endocytic organelles and not, as expected, on the *Toxoplasma* vacuole arising completely separate from the endocytic pathway (Sibley, 2003).

### *Leishmania* Arp2/3 complex genes

**Figure 4:**
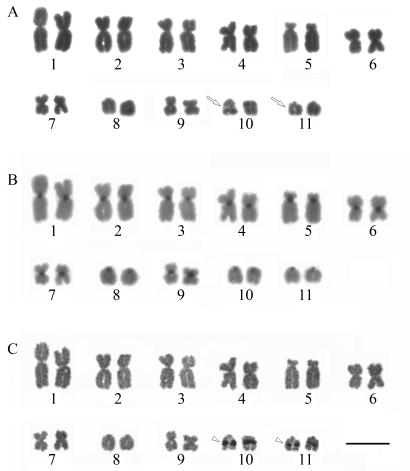
Two dimensional gel electrophoresis of *Leishmania amazonensis* flagellar fraction.  Proteomic analysis after visualization by silver staining. Imaging covering narrow range IPG strips of pH 3-10 - observed in the gel top.  The standard of molecular mass range used is 14400 Da to 97000 Da; -1 is a software configuration parameter.  Six (06) putative spots for coronin are circled and indicated by a red arrow; four (04) Arp2/3 putative spots are circled and pointed by a yellow arrow. The molecular weight (in KDa) and the corresponding isoeletric point of each protein are shown in white.

Arp2/3 complex has emerged as a central effector of actin assembly (Higgs and Pollard, 2001; Humphries *et al.*, 2002). It is composed of seven evolutionarily conserved subunits: two actin-related proteins (Arp2 and Arp3) and five other subunits (yeast Arc40, Arc35, Arc18, Arc19, and Arc15). Since infection of mammalian hosts with *Leishmania* protozoa does depend on the ability of these parasites to replicate within macrophage phagolysosomes, it is noteworthy to link the function of Arps, such as the multiprotein Arp2/3 complex, and the phagosome formation, as a key step in parasite persistence within the host. Previously we have catalogued a list of *Leishmania Arp2/3* complex genes comprising six putative sequences in *L. braziliensis* and *L. infantum*, and five in *L. major*(Costa *et al.*, 2007), which are highly homologous with well-characterized Arp2/3 proteins in different organisms (as seen in Figure 4a). In Figure 4b we show a panel of four modeled Arp2/3 proteins in *Leishmania* where all of them possess the WDR, but no other known binding sites. WDR domains dominantly interfere with coronin function, acting as multimolecular scaffolding domains by bringing together interacting proteins on a single surface (Oku *et al.*, 2003; Yan *et al.*, 2005). They are likely to interfere with normal cell function by scavenging interacting proteins away from endogenous, full-length coronin.

If Arp2/3 complex is, in fact, one of those proteins that interact with coronin (Humphries *et al.*, 2002; Yan *et al.*, 2005), other portions (except WDR) of the protein are responsible for coronin recruitment to sites of actin remodeling, which would, then, link coronin and Arp2/3 to the sites of dynamic actin remodeling, with the contradiction that the WDR domain of coronin would act in a dominant-negative manner by preventing Arp2/3 accumulation. Conflicting interpretations (Humphries *et al.*, 2002; Yan *et al.*, 2005) pose a likely, unforeseen alternative: that coronin might play both positive (stimulating) and negative (inhibiting) roles in actin polymerization, depending on whether Arp2/3 functions prevent branching of F-actin or not.

Since our flagellar proteomic analyses on *L. amazonensis* did demonstrate clear support for the presence of at least four Arp2/3 proteins, as shown in Figure 3, we put forward an interesting point of view regarding the possibility that different sources of Arp2/3 proteins, whether flagellar or from the cytoskeleton, might be responsible in part for such paradox on activities of the coroninArp2/3 interaction.

###  Some *Leishmania* flagellar AIPs are homologous to phagosome proteins

**Figure 5:**
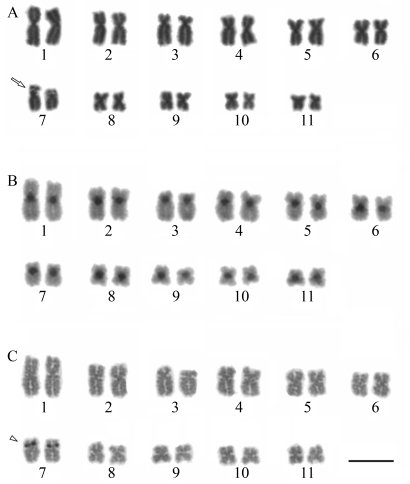
Schematic illustration of *Leishmania spp*. proteins that are seemingly homologous (by sequence alignments) to the proteins of the virtual phagosome depicted on the article by Garin *et al.*, 2001. Selected proteins were used in the present study in an attempt to suggest their potential interaction with phagosomes. Assigned localization to the lumen, the membrane, or the cytoplasmic aspect of the phagosome was the same indicated by Garin *et al.* (2001).

To find out which AIPs in *Leishmania* were homologous to known phagosome proteins, as characterized in other studies (Desjardins *et al.*, 1994; Garin *et al.*, 2001; Marion *et al.*, 2005; Stuart *et al.*, 2007; Haas, 2007), we have searched lists of reported *in vitro* phagosome assays that gave us a panel ranging from 140-617 proteins (Garin *et al.*, 2001; Gotthardt *et al.*, 2006; Stuart *et al.*, 2007) that are studied due to their direct involvement in phagosome formation. Therefore, we have compared their data to that found in *Leishmania* genomes, proteomes and particularly in our *L. amazonensis* flagellar proteome. We then collected a selected list of *Leishmania* proteins, including coronin and Arp2/3, ascribed as highly homologous to mammalian phagosome proteins (Table 1). We built a chart to emphasize these comparisons (Figure 5), which is a modified view of the full virtual phagosome (Garin *et al.*, 2001) to cope with our predictive analysis of parasite flagellar proteins as homologous to mammalian phagosome counterparts. Therefore, in this study, we attempt to make a small contribution to further comprehensive analysis of *Leishmania*-containing phagosomes from which most of proteins have yet to be identified.

Many groups are currently looking into these various aspects of phagocyte biology and progress is steady, although no comprehensive phagosome proteome or lipidome has been published for any pathogen-containing vacuole (Haas, 2007). There is an amazing diversity of compartments that intracellular pathogens inhabit, and they appear to use a stunning number of different factors to establish these compartments. The real challenge remains in understanding how phagocytes and their phagosomes co-operate in pathological situations to phagosome biogenesis, as well as how to turn a phagosome from a hospitable into a killing environment for *Leishmania*.

## Discussion

Phagocytosis is initiated by recognition of a pathogen by a host cell (usually macrophages and neutrophils) receptors that trigger its engulfment into the phagosome. Following their attachment to the macrophage, *Leishmania* promastigotes are internalized to the relatively benign environment of the endosome, where they begin to differentiate into amastigotes. Unlike amastigotes, promastigotes are vulnerable to degradation by the acidic and hydrolytic environment of the phagolysosome (Olivier *et al.*, 2005). They must therefore retard endosome maturation and phagosome-endosome fusion, a process that has been observed by the absence or delayed arrival of late endosomal markers such as rab7 and LAMP-1 (Scianimanico *et al.*, 1999) and is related to the accumulation of F-actin (Holm *et al.*, 2001). The mechanism is not completely understood (Olivier *et al.*, 2005), but it has been shown that the delay in phagolysosome maturation provides a window during which promastigotes can differentiate into the more resistant amastigotes.

Nascent phagosomes have a composition that resembles that of the plasmalemma and are unable to digest the vacuolar contents. This capability is acquired by remodeling of the phagosomal membrane and contents through a complex series of fusion events with compartments of the endocytic and secretory pathways (Desjardins and Descoteaux, 1997). The components delivered to maturing phagosomes include acid hydrolases, as well as vacuolar-type ATPases that are responsible for acidification of the phagosomal lumen. This maturation process culminates in the formation of the phagolysosome, a highly acidic organelle (pH < 5.5) where degradation occurs.

Here we report the association of two classical AIPs (coronins and Arp2/3) to the flagellar compartment in *Leishmania*, certainly a reasonable indication to link flagellar proteins to a possible F-actin binding in this protozoan. *In vitro*, purified coronin is said to bind specifically to F-actin, to bundle actin filaments, and to weakly promote actin assembly (David *et al.*, 1998; Fukui *et al.*, 1999), whereas, in *Acanthamoeba*, coronin was localized in the cell's periphery (on the leading edge) consistent with that of actin located around the phagocytic cups (Baldo *et al.*, 2005). Understanding the molecular mechanisms of phagosome maturation is critical because a number of pathogens, particularly *Leishmania*, survive inside host cells through subversion of this process (Rosenberger and Finlay, 2003). Keeping these facts in mind, together with our own results, we, then, believe that flagellar AIPs, such as coronin and Arp2/3/, might play a role in these subversion mechanisms that maintain the parasite viable within the phagosome.

Some reports have shown that *Leismania* can persist in mammalian neutrophils for up to 48 h, a period that largely exceeds the normal life span of neutrophils, indicating that the phagocytosis/infectious process might alter the ability of neutrophils to initiate their programmed cell death, increasing the lifetime of neutrophils by delaying apoptosis (Aga *et al.*, 2002; Gueirard *et al.*, 2007). It seems that *Leishmania* may have co-evolved with their mammalian hosts (Table 1 and Figure 5) to take advantage of phagosomes to establish a privileged niche for the transient parasitism of leukocytes and their subsequent invasion of macrophages, since it has been shown that the delay in phagolysosome maturation provides a window during which promastigotes can differentiate into the more resistant amastigotes (Olivier *et al.*, 2005).

One could expect that phagosomes containing a given type of particle that entered cells simultaneously via the same receptor would behave the same, at least in a single cell. Surprisingly, however, phagosomes formed via the same receptors are found in different chemical states even within the same macrophage (Griffiths, 2004), a notion that is very useful to help our speculations on *Leishmania* AIPs rich content and at the same time homologous to a few phagosome proteins. Each phagosome is an individual entity whose behavior depends on a finite number of stable equilibrium states in its membrane signaling networks (Griffiths, 2004), which would, thus, underline a diversity of available elements to disturb effective phagosome formation.

As stated by Rasmusson and Descoteaux (2004), *L. donovani* promastigotes inhibit phagolysosome biogenesis in a lipophosphoglycan (LPG)-dependent manner, which correlates with an accumulation of periphagosomal F-actin, forming a physical barrier that prevents *L. donovani* promastigote-containing phagosomes from interacting with endocytic vacuoles. Such inhibition of phagosome maturation may constitute a strategy to provide an environment propitious to promastigote-to-amastigote differentiation. Interestingly, some molecules, known to be involved in apoptotic signaling, are thought to accumulate in phagosome membrane microdomains, suggesting that this location might be important to their specific function, such as LPG in a disruptive process in the phagosome (Gueirard *et al.*, 2007), which could imply an involvement in the ability of *Leishmania* to inhibit neutrophil apoptosis and, thus, the posterior macrophage activity that also depends on actin.

Assuming that the driving force for host cell entry involves polymerization of parasite actin and its AIPs, plus the recognized subcellular localization of functional coronins and Arps shown in the phagosome (Bricheux *et al.*, 2000; Asano *et al.*, 2001; Baldo *et al.*, 2005), then we can propose a possible viable role for flagellar coronin and Arp2/3 within phagosome. As Love *et al.* (1998) have shown, periphagosomal actin is rapidly lost when parasites are internalized. These authors have mentioned a higher percentage of *L. major* promastigotes surrounded by actin after experimentally adding parasites to macrophages. These findings suggest a clear binding to F-actin and implicit evidence of intense actin-interacting activity during phagosome formation after host cell entry (promastigote phagocytosis).

It seems quite obvious that intracellular parasites living within the harsh environment of phagocytes have developed strategies that allow them to adapt quickly, escape from first-line defense systems, and inhibit several functions of their host cells (Olivier *et al.*, 2005). One of these strategies might be explained through the complex interactions of the actin system (Sturgill-Koszycki *et al.*, 1996; Tardieux *et al.*, 1998; Ullrich *et al.*, 1999; Vieira *et al.*, 2002; Yan *et al.*, 2005). Coronins were first identified as AIPs, but, although they are crucial to the dynamics of actin filaments and cell movement, their actin-binding sites have been difficult to pin down, which reinforces the significance of computational biology and proteomic predictions that focus on coronin direct ligands, such as Arp2/3. Moreover, establishing relationships of subcellular/organellar localizations, such as those we now report for coronin and Arp2/3 on the flagellum of *L. amazonensis*, will help to clarify unforeseen roles that parasite AIPs might play on phagocytosis and phagosome formation through direct actin polymerization within the flagellar dynamic environment. This is an issue worthy of further *in vitro* examination that remains now as a direct, positive bioinformatics-derived inference to be presented. Research into phagosome biogenesis has flourished in recent years (Haas, 2007) and it will surely lead to a better understanding of *Leishmania* pathogenesis and mechanisms of immune invasion that involve the flagellar apparatus.

## Figures and Tables

**Table 1:** List of selected *Leishmania* actin-interacting proteins (AIPs) that are seemingly homologous to the phagosome proteins, as described by Garin *et al.* (2001). The proteins are presented with respective sequence ID (as on GeneDB), length (in number of amino acids), predicted molecular weight and isoelectric point (pI).

Protein	Organism	Accession number	MW (kDa)	p*I*	Amino acids
Prohibitin	*L.braziliensis*	LbrM34.0070/LbrM16.1230	32.2/30.2	10.0/8.3	292/268
	*L. infantum*	LinJ16.1700/ LinJ35.0170	30.2/32.3	8.3/9.9	268/292
	*L. major*	LmjF35.0070/ LmjF16.1610	32.3/30.2	9.9/8.3	292/268
RAB2	*L.braziliensis*	LbrM30.1540	25.6	8.3	235
	*L. infantum*	LinJ30.2050	25.6	7.3	235
	*L. major*	LmjF30.1710	25.6	7.3	235
RAB7	*L.braziliensis*	LbrM18.0760/ LbrM10.0880	24.0/24.6	5.3/5.0	223/221
	*L. infantum*	LinJ10.1520	24.9	5.4	222
	*L. major*	LmjF10.1170/ LmjF18.0890	24.7/24.1	5.7/5.1	221/223
RAB11B	*L.braziliensis*	LbrM32.1770	24.8	6.6	219
	*L. infantum*	LinJ32.2250	25.2	7.1	224
	*L. major*	LmjF32.1840	25.2	7.1	224
Stomatin	*L.braziliensis*	LbrM05.0940	40.2	8.6	358
	*L. infantum*	LinJ05.1050	39.6	7.8	357
	*L. major*	LmjF05.1040	39.7	8.3	357
Ubiquitin C	*L.braziliensis*	LM24.42/ LbrM25.0230	34.5/25.1	4.7/5.3	307/233
		LbrM32.0660	26.2	4.5	233
	*L. infantum*	LM24.42/ LinJ32.0850	34.3/26.3	4.9/4.3	307/234
		LinJ25.0190	25.1	4.7	233
	*L. major*	LM24.42 / LmjF32.0700	34.4/26.4	4.8/4.4	307/234
Ubiquitin C	*L.major*	LmjF25.0190	25.1	4.9	233
14-3-3	*L.braziliensis*	LbrM35_V2.3430/LbrM11_V2.0040	29.6/29.0	4.6/4.9	258/253
	*L.infantum*	LinJ36_V3.3360/ LinJ11_V3.0350	29.7/29.2	4.6/4.9	258/253
	*L.major*	LmjF36.3210/ LmjF11.0350	29.6/29.1	4.6/5.0	258/253
ARP3	*L.braziliensis*	LbrM15_V2.1360	38.1	5.4	348
	*L.infantum*	LinJ15_V3.1410	43.7	6.1	405
	*L.major*	LmjF15.1360	43.7	5.9	405
Ash	*L.infantum*	LinJ30_V3.3620	18.6	5.7	162
	*L.major*	LmjF30.3560	18.5	6.1	162
	*L.braziliensis*	LbrM30_V2.3590	18.5	6.9	162
Calreticulin	*L.infantum*	LinJ31_V3.2670	45.0	4.4	400
	*L.major*	LmjF31.2600	45.0	4.4	400
	*L.braziliensis*	LbrM31_V2.2940	48.4	4.1	422
Cathepsin L	*L.major*	LmjF08.1030/ LmjF08.1040	48.0/37.7	7.6/7.1	443/348
		LmjF08.1010/ LmjF08.1020	37.9/37.7	7.3/6.7	348/348
		LmjF08.1050/ LmjF08.1060	37.8/48.0	6.7/7.6	348/443
		LmjF08.1070/ LmjF08.1080	37.8/48.0	6.7/7.6	348/443
	*L.infantum*	LinJ08_V3.0960/ LinJ08_V3.0950	47.9/41.3	7.2/7.5	443/381
	*L.braziliensis*	LbrM08_V2.0810/ LbrM08_V2.0820	47.9/47.9	6.0/7.1	441/441
		LbrM08_V2.0830	47.9	6.7	441
Coronin	*L.infantum*	LinJ23_V3.1400	56.2	7.2	510
	*L.major*	LmjF23.1165	56.6	6.6	510
	*L.braziliensis*	LbrM23_V2.1260	57.0	6.9	510
Cytochrome P450	*L.braziliensis*	LbrM20_V2.2920/LbrM34_V2.2490	61.7/91.3	7.8/8.0	546/832
		LbrM20_V2.2230/ LbrM27_V2.0100	68.4/67.2	6.2/8.9	624/592
		LbrM30_V2.3580	58.0	7.3	509
	*L.infantum*	LinJ35_V3.2600/ LinJ34_V3.3110	91.1/61.3	8.0/6.7	832/546
		LinJ34_V3.3610/ LinJ34_V3.2500	22.8/67.9	10.1/5.6	197/624
		LinJ27_V3.0090	66.7	8.6	592
	*L.major*	LmjF27.0090/ LmjF35.2560	68.5/90.9	8.1/7.9	606/831
		LmjF34.3330/ LmjF30.3550	59.9/58.0	7.6/7.1	533/508
		LmjF34.2670	68.0	6.1	624
HSP-60	*L.braziliensis*	LbrM32_V2.2030/ LbrM30_V2.2790	64.3/58.2	6.0/5.4	594/539
		LbrM35_V2.2240/ LbrM35_V2.2250	60.2/59.5	5.3/5.1	564/562
	*L. infantum*	LinJ32_V3.1940/ LinJ36_V3.2130	64.3/60.5	6.4/5.1	594/566
		LinJ36_V3.2140/ LinJ30_V3.2830	59.3/58.1	5.1/5.2	562/538
	*L. major*	LmjF32.1850/ LmjF36.2020	64.3/60.1	6.3/5.2	594/565
		LmjF36.2030/ LmjF30.2820	59.3/58.0	5.1/5.3	562/538
Prohibitin	*L.braziliensis*	LbrM34.0070/LbrM16.1230	32.2/30.2	10.0/8.3	292/268
	*L. infantum*	LinJ16.1700/ LinJ35.0170	30.2/32.3	8.3/9.9	268/292
	*L. major*	LmjF35.0070/ LmjF16.1610	32.3/30.2	9.9/8.3	292/268
RAB2	*L.braziliensis*	LbrM30.1540	25.6	8.3	235
	*L. infantum*	LinJ30.2050	25.6	7.3	235
	*L. major*	LmjF30.1710	25.6	7.3	235
RAB7	*L.braziliensis*	LbrM18.0760/ LbrM10.0880	24.0/24.6	5.3/5.0	223/221
	*L. infantum*	LinJ10.1520	24.9	5.4	222
	*L. major*	LmjF10.1170/ LmjF18.0890	24.7/24.1	5.7/5.1	221/223
RAB11B	*L.braziliensis*	LbrM32.1770	24.8	6.6	219
	*L. infantum*	LinJ32.2250	25.2	7.1	224
	*L. major*	LmjF32.1840	25.2	7.1	224
Stomatin	*L.braziliensis*	LbrM05.0940	40.2	8.6	358
	*L. infantum*	LinJ05.1050	39.6	7.8	357
	*L. major*	LmjF05.1040	39.7	8.3	357
Ubiquitin C	*L.braziliensis*	LM24.42/ LbrM25.0230	34.5/25.1	4.7/5.3	307/233
		LbrM32.0660	26.2	4.5	233
	*L. infantum*	LM24.42/ LinJ32.0850	34.3/26.3	4.9/4.3	307/234
		LinJ25.0190	25.1	4.7	233
	*L. major*	LM24.42 / LmjF32.0700	34.4/26.4	4.8/4.4	307/234
Ubiquitin C	*L.major*	LmjF25.0190	25.1	4.9	233
14-3-3	*L.braziliensis*	LbrM35_V2.3430/LbrM11_V2.0040	29.6/29.0	4.6/4.9	258/253
	*L.infantum*	LinJ36_V3.3360/ LinJ11_V3.0350	29.7/29.2	4.6/4.9	258/253
	*L.major*	LmjF36.3210/ LmjF11.0350	29.6/29.1	4.6/5.0	258/253
ARP3	*L.braziliensis*	LbrM15_V2.1360	38.1	5.4	348
	*L.infantum*	LinJ15_V3.1410	43.7	6.1	405
	*L.major*	LmjF15.1360	43.7	5.9	405
Ash	*L.infantum*	LinJ30_V3.3620	18.6	5.7	162
	*L.major*	LmjF30.3560	18.5	6.1	162
	*L.braziliensis*	LbrM30_V2.3590	18.5	6.9	162
Calreticulin	*L.infantum*	LinJ31_V3.2670	45.0	4.4	400
	*L.major*	LmjF31.2600	45.0	4.4	400
	*L.braziliensis*	LbrM31_V2.2940	48.4	4.1	422
Cathepsin L	*L.major*	LmjF08.1030/ LmjF08.1040	48.0/37.7	7.6/7.1	443/348
		LmjF08.1010/ LmjF08.1020	37.9/37.7	7.3/6.7	348/348
		LmjF08.1050/ LmjF08.1060	37.8/48.0	6.7/7.6	348/443
		LmjF08.1070/ LmjF08.1080	37.8/48.0	6.7/7.6	348/443
	*L.infantum*	LinJ08_V3.0960/ LinJ08_V3.0950	47.9/41.3	7.2/7.5	443/381
	*L.braziliensis*	LbrM08_V2.0810/ LbrM08_V2.0820	47.9/47.9	6.0/7.1	441/441
		LbrM08_V2.0830	47.9	6.7	441
Coronin	*L.infantum*	LinJ23_V3.1400	56.2	7.2	510
	*L.major*	LmjF23.1165	56.6	6.6	510
	*L.braziliensis*	LbrM23_V2.1260	57.0	6.9	510
Cytochrome P450	*L.braziliensis*	LbrM20_V2.2920/LbrM34_V2.2490	61.7/91.3	7.8/8.0	546/832
		LbrM20_V2.2230/ LbrM27_V2.0100	68.4/67.2	6.2/8.9	624/592
		LbrM30_V2.3580	58.0	7.3	509
	*L.infantum*	LinJ35_V3.2600/ LinJ34_V3.3110	91.1/61.3	8.0/6.7	832/546
		LinJ34_V3.3610/ LinJ34_V3.2500	22.8/67.9	10.1/5.6	197/624
		LinJ27_V3.0090	66.7	8.6	592
	*L.major*	LmjF27.0090/ LmjF35.2560	68.5/90.9	8.1/7.9	606/831
		LmjF34.3330/ LmjF30.3550	59.9/58.0	7.6/7.1	533/508
		LmjF34.2670	68.0	6.1	624
HSP-60	*L.braziliensis*	LbrM32_V2.2030/ LbrM30_V2.2790	64.3/58.2	6.0/5.4	594/539
		LbrM35_V2.2240/ LbrM35_V2.2250	60.2/59.5	5.3/5.1	564/562
	*L. infantum*	LinJ32_V3.1940/ LinJ36_V3.2130	64.3/60.5	6.4/5.1	594/566
		LinJ36_V3.2140/ LinJ30_V3.2830	59.3/58.1	5.1/5.2	562/538
	*L. major*	LmjF32.1850/ LmjF36.2020	64.3/60.1	6.3/5.2	594/565
		LmjF36.2030/ LmjF30.2820	59.3/58.0	5.1/5.3	562/538

*Predicted Molecular Weight and Isoeletric Point (pI). 14-3-3 proteins are a family of conserved regulatory molecules expressed in all eukaryotic cells. 14-3-3 proteins have the ability to bind a multitude of functionally diverse signaling proteins, including kinases, phosphatases, and transmembrane receptors, being considered evolved members of the Tetratricopeptide Repeat (TPR) superfamily. *Predicted features and general scheme are the same as at GeneDB, including accession number / ID (Lmj = *L. major*; Lin = *L. infantum*; Lbr = *L.braziliensis*), according to the manuscript text.
